# Recent Advances in the Carotenoids Added to Food Packaging Films: A Review

**DOI:** 10.3390/foods12214011

**Published:** 2023-11-02

**Authors:** Swarup Roy, Ram Kumar Deshmukh, Shefali Tripathi, Kirtiraj K. Gaikwad, Sabya Sachi Das, Devanshi Sharma

**Affiliations:** 1 Department of Food Technology and Nutrition, School of Agriculture, Lovely Professional University, Phagwara 144411, Punjab, India; 2 Department of Paper Technology, Indian Institute of Technology Roorkee, Roorkee 247667, Uttarakhand, India; rkdeshmukh9421@gmail.com (R.K.D.); shefali_t@pt.iitr.ac.in (S.T.); kirtiraj.gaikwad@pt.iitr.ac.in (K.K.G.); 3 School of Pharmaceutical and Population Health Informatics, DIT University, Dehradun 248009, Uttarakhand, India; sabya2049@gmail.com; 4 Institute of Science, Nirma University, SG Highway, Ahmedabad 382481, Gujrat, India; devanshisharma953@gmail.com

**Keywords:** carotenoids, packaging, functional, food shelf life, biomaterials, sensors

## Abstract

Food spoilage is one of the key concerns in the food industry. One approach is the improvement of the shelf life of the food by introducing active packaging, and another is intelligent packaging. Detecting packed food spoilage in real-time is key to stopping outbreaks caused by food-borne diseases. Using active materials in packaging can improve shelf life, while the nonharmful color indicator can be useful to trace the quality of the food through simple color detection. Recently, bio-derived active and intelligent packaging has gained a lot of interest from researchers and consumers. For this, the biopolymers and the bioactive natural ingredient are used as indicators to fabricate active packaging material and color-changing sensors that can improve the shelf life and detect the freshness of food in real-time, respectively. Among natural bioactive components, carotenoids are known for their good antimicrobial, antioxidant, and pH-responsive color-indicating properties. Carotenoids are rich in fruits and vegetables and fat-soluble pigments. Including carotenoids in the packaging system improves the film’s physical and functional performance. The recent progress on carotenoid pigment-based packaging (active and intelligent) is discussed in this review. The sources and biological activity of the carotenoids are briefly discussed, and then the fabrication and application of carotenoid-activated packaging film are reviewed. The carotenoids-based packaging film can enhance packaged food’s shelf life and indicate the freshness of meat and vegetables in real-time. Therefore, incorporating carotenoid-based pigment into the polymer matrix could be promising for developing novel packaging materials.

## 1. Introduction

Nowadays, food safety and food spoilage are serious concerns. Food-borne outbreaks are often caused worldwide due to the growth of unwanted food-poisoning pathogens. To deal with this issue, active and intelligent packaging is useful. Active packaging contains a bioactive functional ingredient that helps improve the packaging material’s antioxidant, amicrobial, or UV-light barrier performance. One of the other ways to prevent food poisoning is by sensing the condition of food through smart packaging. Smart packaging helps indicate packed food items’ condition in real-time. It is intelligent indicator-based packaging in which a pH indicator, gas sensor, or time temperature indicator is included in the packaging system [1,2]. Active packaging adds functionality to the packaging material, which can restrict the growth of food-borne pathogens and limit the oxidation of food.

In contrast, intelligent packaging provides information on spoilage, temperature change, and food safety to consumers without opening the packed food item from the container [3,4]. Compared to its competitors, pH indicator-based intelligent packaging is the most basic yet accurate, cost-effective, user-friendly, and non-destructive technology [5,6,7,8]. The pH-changing film with added pigment changes color depending on the food’s condition [9,10]. Alteration in the pH of perishable food, such as meat, fish, etc., is common during storage due to food spoilage by oxidation or microbial contamination [11,12,13,14,15,16]. If the pH of the food either decreases or increases during storage, it can easily be identified using a packaging color indicator.

Recently, many reports have been published on active packaging and pH-sensitive color indicator-based intelligent packaging. The synthetic chemicals used as active fillers in food packaging have serious health consequences; thus, the natural bioactive ingredients are superior to their chemical counterparts. The addition of bioactive ingredients to the packaging can be advantageous to strengthen the shelf life of food. In the case of smart packaging, synthetic dyes like bromocresol green, methyl red, etc. are used. Still, owing to this commercial dye’s health and environmental toxicity, the safer alternative is natural pigments such as anthocyanin, curcumin, betalains, carotenoids, etc. [17,18,19,20,21,22]. Many reports on intelligent packaging indicators using anthocyanin and curcumin are available due to their widespread availability and good color-indicating properties. Carotenoids are also used as a color indicator in intelligent packaging materials. This natural pigment is fat-soluble and known for excellent pH-responsive color change, thus evolving as a potential candidate for making smart food freshness tracing indicators [23,24,25]. Carotenoids are tetraterpene pigments highly abundant in fruits and vegetables, and they exhibit various colors like yellow, red, purple, etc. [26,27,28]. Many bioactive components exist in carotenoids, such as beta-carotene, norbixin, zeaxanthin, lycopene, etc. [27,29,30,31]. These bioactive components of carotenoids are strong antioxidant and antimicrobial agents and, thus, potential candidates for active packaging ingredients.

Several reports have recently been published on carotenoid-based active and intelligent packaging [30,32,33,34,35,36,37] and, as far as we know, only a few reports have comprehensively summarized the potential carotenoids-based pigments for manufacturing active and intelligent food packaging material. Recently, the potential of carotenoids in active and intelligent packaging was reviewed [38,39]. Thus, this review aims to provide an overview of carotenoids, their significance, various sources, and important functional properties. Moreover, the fabrication of carotenoid-activated packaging film is also discussed, and finally, carotenoid-based intelligent packaging and its application in food are debated. This review is expected to increase interest among researchers in exploring the potential of carotenoids in active and intelligent food packaging applications [40,41,42,43].

## 2. Carotenoids and Their Importance

Carotenoids are naturally occurring chemicals found in a wide range of natural sources. Photosynthetic organisms can produce carotenoids, including cyanobacteria, algae, plants, certain fungi, and bacteria. In contrast, most animals cannot generate carotenoids, although they can receive them through nutrition and structural changes. However, certain invertebrates, such as hemipteran insects (e.g., aphids, adelgids, and phylloxera), dipteran insects (e.g., gall midges), and mites, have the unique ability to synthesize carotenoids from scratch [44]. The name “carotenoids” refers to a large classification of pigments in both the animal and plant worlds. This class of fat-soluble pigments includes about 700 chemicals that produce red, orange, and yellow color in various organisms [45]. Furthermore, carotenoids can be produced by some non-photosynthetic bacteria and fungi [46].

Carotenoids’ high level of unsaturation makes them sensitive to trans-isomerization when exposed to stimuli such as heat, light, and acids. Carotenoids are the most stable structure seen in nature in their trans-form. Exposure to these stimuli, on the other hand, can cause a change to the less stable cis-form, resulting in a little loss of color and provitamin activity. Carotenoids are also susceptible to oxidation, which can occur through enzymatic or nonenzymatic mechanisms. Furthermore, high temperatures and light exposure can influence carotenoids’ susceptibility to oxidation. Carotenoids are hydrophobic substances, which means they are not soluble in water. Instead, they are lipophilic, dissolving easily in lipids and other non-polar solvents. Carotenoids are thus insoluble in water but highly soluble in organic solvents such as acetone, alcohol, and chloroform.

**Figure 1:**
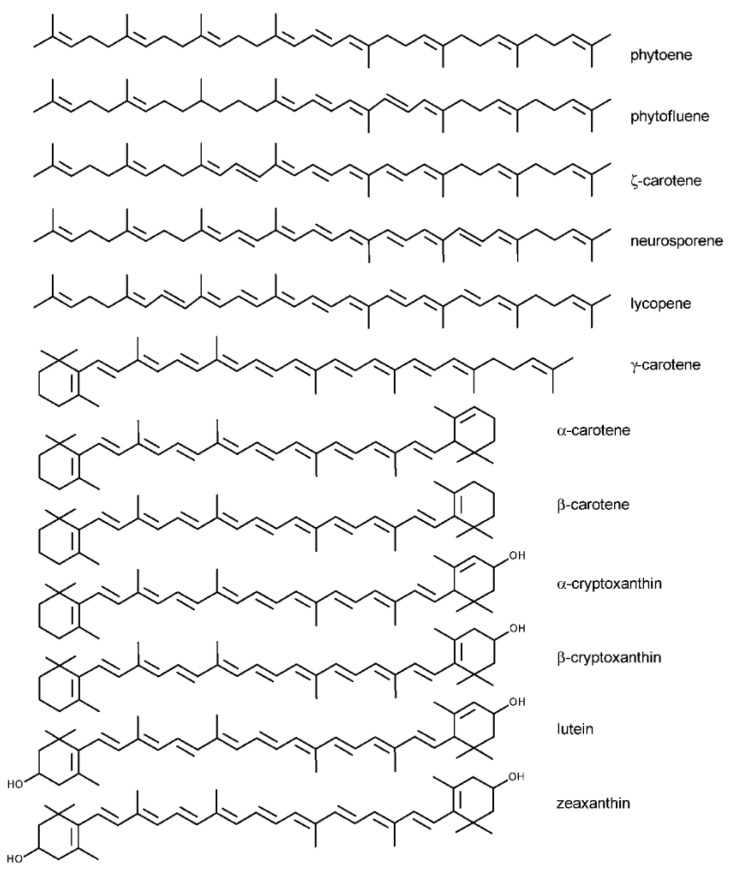
Chemical structures of major carotenoids [47].

Carotenoids are categorized into two types in nature (Figure 1): (a) Carotenes: One example is *β*-carotene, which is made up of linear hydrocarbons that can be cyclized at one or both ends of the molecule. (b) Xanthophylls: These are carotenoid oxygenated derivatives that include lutein, violaxanthin, neoxanthin, and zeaxanthin. The presence of oxygen-containing functional groups in the chemical structure of xanthophylls distinguishes them [46]. The bulk of carotenoids are tetraterpenoids (C40), which have 40 carbon atoms. These carotenoids are composed of eight isoprenoid units connected in a linear and symmetrical pattern, with the order reversed in the center. The basic cyclic structure can be modified through hydrogenation, dehydrogenation, cyclization, and oxidation. These pigments have a high chemical reactivity due to a conjugated system of double bonds, rendering them susceptible to simple isomerization and oxidation reactions.

## 3. Sources of Carotenoids

A large variety of regularly grown and eaten fruits contain carotenoids. Citrus fruits, mangoes, papayas, apricots, and peaches, among others, are well-known sources of carotenoids. Several vegetables, in addition to fruits, are great sources of carotenoids [48]. Green foods such as spinach, broccoli, carrots, red peppers, and tomatoes are commonly consumed vegetables high in carotenoids. Including these fruits and vegetables in your diet will help you get a good dose of carotenoids, which have a variety of health benefits due to their antioxidant characteristics and provitamin A activity [48,49].

Carotenoid content analyses of underutilized, non-domesticated, and alien plant foods have been a contemporary research focus. This work is still very relevant and significant. Researchers and scientists are increasingly interested in studying the carotenoid composition of less typically consumed plant foods, particularly those not commonly consumed in the domesticated or mainstream diet [50,51]. Lutein and zeaxanthin have been found in various plant foods, including sastra, corozo, and sapote. These carotenoids are well-known for preserving eye health and lowering the risk of age-related macular degeneration.

On the other hand, lycopene has been found in various plants, including sarsaparilla and buffaloberry. Lycopene is well-known for its significant antioxidant capabilities, which may aid in preventing some malignancies and support cardiovascular health [52,53]. Carotenoids are natural colors that are abundant in nature. They can be found in various living creatures, including plants and microbes. Carotenoids are biosynthesized in these species and play important roles, such as collecting light during photosynthesis and guarding against oxidative stress [54]. Many carotenoids utilized in many industries, particularly industrial pigments and dietary supplements, are chemically synthesized. For example, astaxanthin, a red pigment and potent antioxidant, is widely chemically synthesized for industrial applications. Another carotenoid utilized as a food and feed supplement, canthaxanthin, is generally synthesized through chemical synthesis.

Furthermore, to suit market demand, *β*-carotene, a well-known carotenoid with provitamin A activity, is synthetically created. Concerns about food safety, environmental impact, and the potential negative effects of chemically synthesized carotenoids have fueled interest in researching natural alternatives for carotenoid production. Natural and eco-friendly solutions are becoming increasingly popular as customers become more concerned about the origin and safety of their food [55,56].

**Figure 2:**
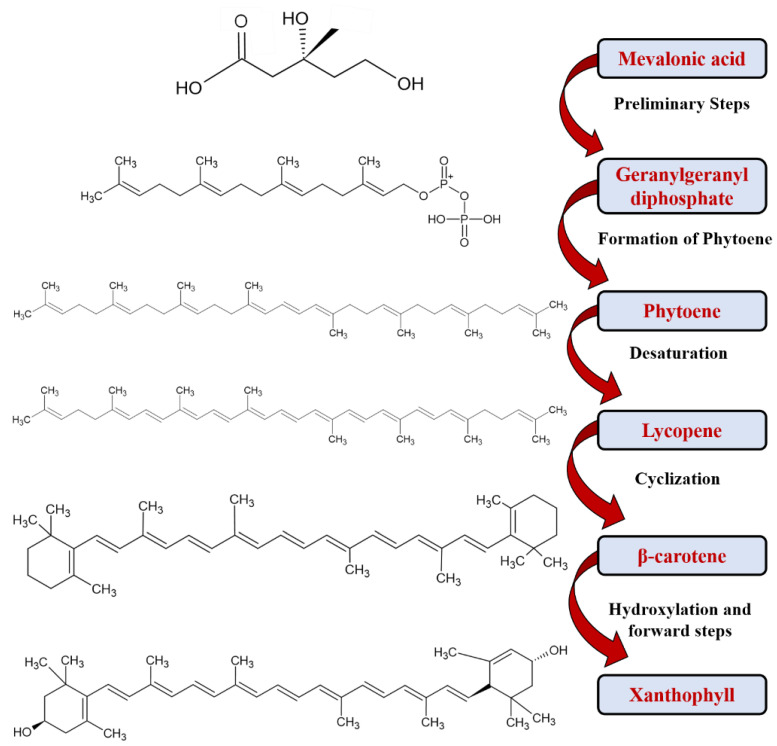
Scheme representing major steps involved in the biosynthesis of carotenoids [58].

The bioproduction of carotenoids (Figure 2) is increasing due to various causes. These criteria include low-cost substrates, natural chemicals, and small production regions. Furthermore, bioproduction is unaffected by environmental factors such as climate, season, or soil composition, and it provides greater control over growing conditions [57]. Carotenoids are produced by both photosynthetic organisms (such as algae and cyanobacteria) and non-photosynthetic microbes (such as bacteria, fungi, and yeasts). The carotenoid production by the biotechnological process has been widely investigated, dethatching the commercial production of carotenoid types from microorganisms found in various sources. Technologically interesting microorganisms exhibit high potential in the bioproduction of various carotenoids and their byproducts (Table 1).

## 4. Biological Activity of Carotenoids

Carotenoids are widely used in food and are frequently used as antioxidants and natural colorants. Carotenoids rich in provitamin A can be found in dark green and yellow-orange leafy foods. The intensity of color in these vegetables is related to their provitamin A concentration. Carotenoids are superb antioxidants, scavenging and neutralizing free radicals, as demonstrated in in vitro and in vivo studies. The carotenoids are also known for their antimicrobial activity against broad-spectrum pathogens.

Carotenoids are naturally occurring pigments that play a crucial role in protecting plants against photooxidative processes. They exert potential antioxidative activity by scavenging various reactive oxygen species (ROS), including singlet oxygen and peroxyl radicals [76,77]. The bioactive components of carotenoids, such as beta-carotene, norbixin, zeaxanthin, lycopene, etc., are known for their strong antioxidant activity [78,79,80].

The major functional activity of carotenoids is attributed to their double-bond (conjugated) structural arrangements. Discussing and highlighting these molecules’ biological effects, such as antimicrobial activity, becomes significant in establishing the precise mechanism for clinical interest [81]. Various quantitative and qualitative studies have been performed to assess the antimicrobial potential of crude extracts composed of carotenoids and other metabolites. The carotenoids were extracted from various *Rhodotorula* spp. (*Rhodotorula mucilaginosa, Rhodotorula glutinis*, and *Rhodotorula rubra*), where torularhodin and torulene were found as major carotenoids. It was observed that only one study was tested and resulted in bacterial wild strains attained from pig semen [82]. In contrast, four reports were primarily focused on *Candida* spp. [83,84]. Studies have suggested potential applications of *Rhodotorula* spp., helpful in carotenoid synthesizing, as an alternate source of natural preservers (antimicrobial and antioxidant), pigments (colorants), and nutraceuticals. A study observed that carotenoids extracted from various *Rhodotorula glutinis* strains exhibited potential antibacterial and antioxidant activity [85]. In a study, the researchers evaluated the antibacterial activity of carotenoid-bacterium symbionts (*Virgibacillus salarius*-*Sinularia* sp.) against the growth of MDR *Escherichia coli* and methicillin-resistant *Staphylococcus aureus* (MRSA). The statistical analysis showed significant differences between the concentration, carotenoid pigment, and positive control groups [86].

Fucoxanthin (FXT), a carotenoid produced by diatoms and brown algae, has shown numerous biological activities, including antioxidant, antimicrobial, and others. Karpiński and Adamczak, in their research, demonstrated that the isolated FXT exhibited more significant antibacterial effects against Gram-positive bacterial strains than Gram-negative bacterial strains. Moreover, according to the results of the agar disc-diffusion method, the highest antibacterial activity (higher zone of inhibition) of FXT was exhibited against *Streptococcus agalactiae*, *Staphylococcus epidermidis*, and *Staphylococcus aureus* [87]. In another study, FXT isolated from *Turbinaria triquetra* showed potential antibacterial activity against both Gram-positive and Gram-negative bacteria within a concentration range of 10–100 µg/mL [88]. FXT, in higher concentrations, showed significant antimicrobial activity against *Listeria monocytogenes*, *Staphylococcus aureus*, *Enterococcus* sp., *Bacillus subtilis*, and *Psuedomonas aeruginosa* [89]; however, higher concentrations of phytoconstituents may cause toxicity.

**Figure 3:**
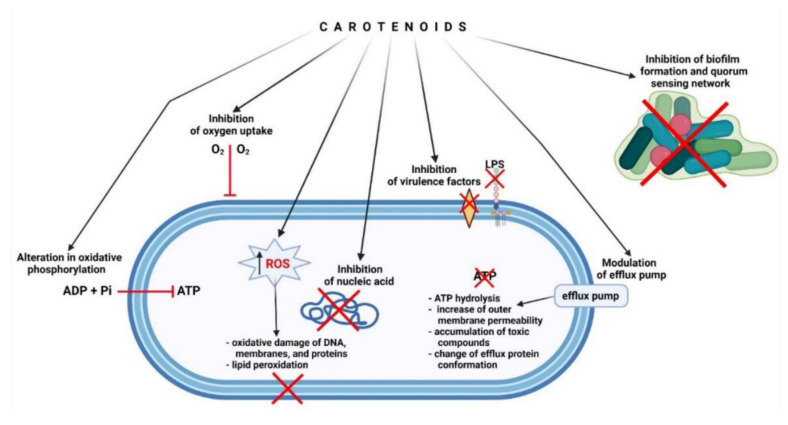
Potential antibacterial mechanisms of action of carotenoids, such as astaxanthin and fucoxanthin. Adapted from [70]. (Reprinted from a Creative Commons Attribution (CC BY) license).

One of the studies detailed the antibacterial activity of an orange-colored pigment formed from *Parococcus homiensis* strain BKA7 identified in the atmospheric air of Basra city, Iraq. Further, it was observed that the extract was composed of carotenoids (β-carotene) and xanthophylls. *β*-carotene exhibited potential antioxidant activity (confirmed through a DPPH assay) and antibacterial activity, more significantly against Gram-positive than Gram-negative bacteria [90]. Natural xanthophylls such as AXT and FXT have been reported to exhibit various pharmacological activities, including antioxidant, antimicrobial, anticancer, anti-inflammatory, and others [70]. The exact antimicrobial mechanisms of xanthophylls are still not revealed; however, they show a similar mechanism (Figure 3) as that of the carotenoids and terpenoids class. Earlier studies have reported that carvacrol (terpenoid) directly affects the bacterial cell, leading to the deterioration of the cell wall and cell membrane and the outflow of cellular constituents [91].

Shanmugapriya et al. formulated AXT-loaded nanoemulsions for treating infections caused by both Gram-positive and Gram-negative bacterial species [92]. In another study, the parasitic (*Trypanosoma cruzi*) load was significantly reduced in vitro, but no significant observation was observed in vivo when studied in a mouse model [93]. In another study, AXT showed a potential antibacterial effect in a *Helicobacter pylori*-infected mouse model. In the same study, AXT exhibited significant anti-inflammatory activity with reduced gastric inflammation and cytokine release through splenocytes. In addition, various countries have already recommended or approved AXT doses between 2 and 24 mg. As per European Food Safety Authority guidelines, AXT’s projected adequate daily consumption is 2 mg [94]. Also, AXT is approved by the United States Food and Drug Administration and is generally recognized as safe (GRAS) for both animals and humans in terms of diet usage [95].

## 5. Fabrication and Characterization of Carotenoid-Based Packaging Films

The carotenoids present a broad class of natural colorants or pigments, antioxidants, and nutraceutical compounds, mostly separated from the different plant and fauna parts. Carotenoids are mostly tetraterpene pigments, observed in different colors such as yellow, orange, red, and purple. In recent years, these carotenoids have attracted academicians and industries to incorporate value addition to foods, preservations, and active and intelligent packaging development [26]. Active packaging supports the longer shelf life of food. In contrast, intelligent packaging helps monitor the condition of food and the package environment for consumers in real-time through the implanted sensors or indicators on the package as a label, which detect unusual changes (e.g., pH, temperature, relative humidity, moisture, and bacteria). Intelligent packaging is based on a color variation on the packaging label, which can be easily detectable by consumers [96].

Indeed, intelligent packaging has emerged as a means of communication between the food in the package and the consumer. It is developed using biopolymers incorporating active compounds from plants as pigment ingredients, such as carotene from carrots, lycopene from tomatoes, curcumins from turmeric, etc. [97]. These carotenoids are responsible for the color development of active and intelligent packaging, which varies as per their antioxidant activity, antimicrobial function, and color-changing mechanism, which could be based on pH variations, temperature, or microorganisms. Along with their intellectual properties, they possess active functional properties, which could be added benefits when incorporating carotenoids from natural sources into the packaging system.

**Figure 4:**
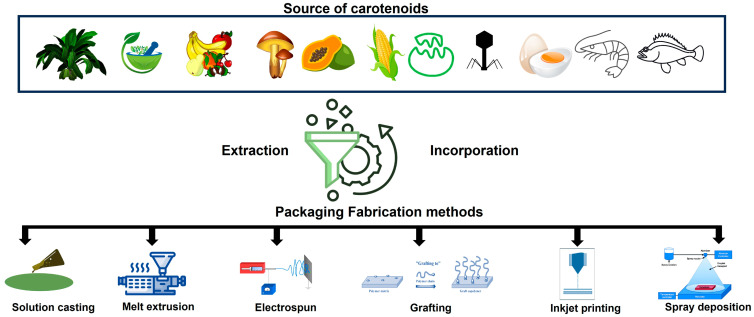
Development of active and intelligent food packaging systems with different methods incorporating carotenoids from different sources.

Incorporating these bioactive functional compounds into the polymeric matrix is still a critical development challenge. The fabrication method of the packaging matrix further explores the possible incorporation of carotenoids in the polymeric matrix or as a label on the package [98]. There are several methods of biopolymer packaging film development, such as solution casting [99], melt extrusion [100], mold compression [101], electro-spinning [102], photo-grafting [103], inkjet printing [104], spraying [105], etc. The carotenoid-based packaging films are mostly developed using solution casting due to convenience and economics at the laboratory level, as illustrated in Figure 4.

### 5.1. Solution Casting

Solution casting is the oldest and most convenient method to develop packaging film and was discovered in the 19th century by Eastman Kodak to make plastic films. It is a versatile process for fabricating thin films or sheets on a small scale or for laboratory experiments. The polymer solutions of different polymers are dissolved in suitable solvents, either aqueous or non-aqueous volatile solvents, occasionally reinforcing various additives such as micro- or nano-sized materials before being cast on the flat surface. Then, the solvent phase is evaporated with different drying methods, and the dried thin film is cast off from the substrate [106]. Incorporating lycopene and β-carotene into the sodium alginate composite improved functional properties at 0.1%, 0.3%, and 0.5%. In addition, the carotenoid thickness of the film was unaffected; however, the tensile properties of the composite were found to be significantly varied even at lower concentrations of carotenoids. The barrier properties to the water vapor reduced significantly (*p* < 0.05) at 0.5% of carotenoids, and light transmission improved along with the thermal stability of the film [107]. In another study, a polylactide (PLA) composite was fabricated by incorporating bixin carotenoids and acetyl tri-butyl citrate (ATBC) and found to have improved light barrier properties. At higher processing temperatures, the mixed carotenoid degraded up to 74%, even though the composite had 95% blocking of UVA and 90% of UVB. The plasticizer ATBC accelerated the carotenoid release into the food simulant, allowing controlled outreach of the carotenoid for food preservation [108]. Solution casting has explored the maximum possibilities with different carotenoid-based packaging films for food applications such as β-carotene, lycopene, xanthophyll, lutein, canthaxanthin, etc.

### 5.2. Melt Extrusion

The raw material is transformed into a substance with a distinct structure and textural qualities throughout the extrusion process. The combination of diverse forces in the extrusion, such as shear, temperature, and pressure, causes the raw material’s melting at low/high moisture content to expand and gelatinize. The liquid phase containing biopolymers is created by extrusion discharged during expansion, facilitating the formation of the molded structure of the film or package container [109]. Incorporating carotenoids into the melt-extrusion method frequently used to create biodegradable packaging materials can offer improved functionality and other advantages [110]. Carotenoids are natural pigments with antioxidant activity and color-changing qualities that can be found in a variety of fruits and vegetables. In the melt extrusion, the carotenoid combination and biodegradable polymer are put into an extruder, which melts and mixes the components at a predetermined temperature and pressure. The extruder consists of a barrel, one or more screws, and a die. A homogenized melt is created by heating and moving the carotenoid–polymer mixture with the screw [111].

In another study, the packaging film was developed with different concentrations of cassava starch, poly (butylene adipate-co-terephthalate) (PBAT), coconut nanocellulose, and citric acid. It was observed that the mechanical strength significantly improved with its elongation from 206.31 to 278.41% for various film combinations. The film’s scanning electron microscope (SEM) analysis predicted that the visible microporous allowed the moisture to diffuse from the product to its surface, which helps maintain the product’s color for a longer duration [112]. In another similar investigation, researchers demonstrated the single-step scalable extrusion of polylactic acid (PLA)/cellulose nanocrystals (CNCs), improving processability and rheological behaviors. The PLA was grafted onto the CNCs, resulting in cross-linked gel-like structures with varying graft efficiency and gel-formation capability depending upon the compatibilizers used. The extruded film showed a reduction in both oxygen (20–65%) and water vapor permeability (27–50%), along with its significantly improved thermomechanical properties [113].

### 5.3. Electrospinning

The assembly of two highly sophisticated methods, electrospray and spinning, is made using electrospinning (electro + spinning). When a high electric field is applied to the melted fluid or solution from the die tip, it also acts as an electrode due to the high field energy. The resulting formation, which comes out as an ejected charged jet from the die tip towards the counter electrode, leads to continuous fiber formation [114]. The production of the thinnest fibers, possibly up to the nanometer range, with a large surface area, superior mechanical properties, and the capability to produce three-dimensional (3D) objects makes it easy to use them for various functional applications. Electrospinning has often been utilized in biomedical and tissue engineering, using natural bioplastics such as proteins, polysaccharides, and lipidic formations [115]. Biopolymers are the preferred choice for active and intelligent packaging for food due to their functional properties, ability to carry active agents, and controlled release. The active and intelligent agents from different natural sources are thermally sensitive and evaporate while drying the incorporated film due to their high volatility. Electrospinning can be an option to overcome this problem with electrospun fiber encapsulation. The above factors increased interest in using electrospun fiber in the food and packaging industries [116]. Electrospun fibers are applicable in the food industry in many ways, including as reinforcement agents for eco-friendly packaging, emulating elements for artificial foods, scaffolding for cell cultures, encapsulation of enzymes, vitamins, and antimicrobials, etc. [117]. Electrospun nanofibers have mechanical properties and can aid the biopolymers in strengthening the functional properties of the packaging films as reinforcing fillers.

Proteins and polysaccharides are commonly used for this purpose; preferably, protein is a major ingredient in the human body [118]. Emulsions were created by mixing SPI and PVA in a 50:50 ratio, combined with soybean oil (SBO) as a carrier for β-carotene. These emulsions were electrospun directly onto a PHA-based film. An annealing process was used to enhance adhesion between the coating and the film and control the release rate of β-carotene. The bioactive electrospun fibers showed high encapsulation efficiency (65.0% ± 2.6%), with 51.4% ± 0.9% of the β-carotene effectively incorporated within their cores. In vitro release assays in soybean oil revealed that annealing resulted in a slower and more sustained release of the bioactive compound [119]. The electrospun gallic acid-loaded zein fiber mat was produced for the potential active food packaging application. The fabricated mat showed low water activity, and the FTIR-ATR and thermogravimetric analysis revealed the mat’s stability over time. Gallic acid followed the Fickian diffusion method, and the data fit better with the Higuchi model. The fabricated mat showed better antibacterial activity and the desired functional properties for active food packaging applications. SPI and PVA were blended in a 50:50 ratio with soybean oil (SBO), serving as a vehicle for carotene to generate emulsions. These emulsions were electrospun directly onto a PHA-based film.

An annealing procedure was employed to slow the release of beta-carotene and improve adhesion between the coating and the film. The bioactive electrospun fibers demonstrated significant encapsulation efficiency (65.0% ± 2.6%), efficiently incorporating 51.4% ± 0.9% of the beta-carotene inside their cores. Annealing caused the bioactive ingredient to release more gradually and steadily, according to in vitro release tests in soybean oil [119]. The research described the electrospinning of -Car/HP-CD and -Car/HP-CD nanofibers without using polymers from aqueous and organic solutions. The phase solubility technique showed that lipophilic -Car could be solubilized in water by both HP- and HP-CD, displaying a 1:1 M solubility of -Car by CDs. Bead-free fibers were created from aqueous and organic solutions using the right amounts. The effective integration of -Car into CD fibers was confirmed by FTIR analysis. XRD and dissolving tests confirmed that CDs and -Car formed an inclusion complex. Molecular modeling studies showed that HP-CD and -Car may form a 1:1 inclusion complex and a 1:2 inclusion complex, respectively. Due to the stabilizing impact of CD molecules on the entrapped -Car, the inclusion of complex nanofibers displayed excellent antioxidant activity, and unlike -Car alone, they were stable during UV exposure. Using the inherent activity of -Car, theater-borne electrospinning of -Car and HP–CD/HP–CD proposes that these antioxidant materials may find widespread uses in the food and biomedical areas [120].

### 5.4. Grafting-Polymerization

In recent years, biopolymers from natural sources have proved miraculously useful in food and pharmaceutical applications as diluents, binders, gelling agents, and thickeners for colloidal suspension. Currently, it does not show the necessary properties required to fulfill different applications, so modifying the polymer at the chemical functional group is required to lead to a wide range of favorable improvements and refinements. Reactive functional groups such as hydroxyl, thiol, carboxylic acid, and amino groups indicate the possible sites for chemical modification utilizing grafting [121]. Grafting is a method where monomers are covalently reinforced onto the polymeric chain. Primarily, grafting is performed to enhance biocompatibility, wettability, and mechanical properties. Synthetic, radiation, photochemical, enzymatic, and plasma-actuated grafting strategies are imperative for polymer modification. Photographing monomers onto a conventional packaging polymer fabricated a non-migratory antioxidant clean label. Using the photographing technique to create a reactive surface with a broader oxirane ring, glycidyl methacrylate (GMA) was brushed over the polypropylene (PP) film. To develop an antioxidant film, a conducting polymer was anchored to the surface of the reactive film through a ring-opening process. Numerous studies on food preservation showed that antioxidant films might increase shelf life and even slow ascorbic acid breakdown [122]. Another experiment used images to create a ligand film with citric acid as a chelator on the surface. The polypropylene film was brushed photochemically by immobilizing glycidyl methacrylate and benzophenone on the film’s surface. Using FESEM and atomic force microscopy (AFM), the microstructure, grafting morphology, and spiral structure with a porous surface were examined. This film demonstrated significant chelating action in virgin olive oil and vitamin C (*p* < 0.05), prolonging the shelf life of both goods [123].

### 5.5. Inkjet Printing

Inkjet printing is an efficient, flexible, cost-effective, and versatile method for development, with a non-contact method for manipulating the indicator composition for intelligent packaging systems to enhance detection sensitivity/precision [124]. Inkjet printing could be based on thermal and piezoelectric technologies. The different methods of ink ejection are based on the pressure generated from vapor bubble expansion in thermal inkjet printing, while the piezoelectric inkjet method involves the physical deformation of material from the nozzle through an applied pulse electric field. Typical substrates for printing can be paper and polymers based on end-user requirements and convenience [125].

This study demonstrated that inkjet-printed gradient indicators may monitor the freshness of fresh fish fillets by demonstrating substantial color variations over time. The indicators distinguished storage time and variations in volatile amine content. Colorimetric indicators with “best-before” labels might indicate precise freshness. Inkjet printing enables large-scale production and possible mobile device use. Future studies should look at the influence of various packing types, storage circumstances, and perishable items on the color response of the indicator. Avoiding indicator dye migration and investigating additional spoilage metabolites is critical. Naturally occurring food-grade colorants may help alleviate toxicity in direct food contact applications [126]. Another investigation was reported, where a pH-sensitive quick response label (QR code) was developed with inkjet technology. The inks for this label were prepared with roselle anthocyanin and curcumin with an evaluation of rheological, surface tension, and contact angle properties. The QR code printed with different concentrations of different compositions showed sensitivity differences based on the ammonia solution percentage. It was also observed that the color was significantly varied as the ink ratio was changed, which made it possible to apply it for QR code freshness monitoring in real-time. Therefore, this label could be used as a data carrier to deliver comprehensive information about a product, such as an expiration date, composition, manufacturer details, etc. [127].

### 5.6. Coating Deposition

The coating is applied to the thin film or substrate polymer using continuous layer deposition. These coatings work as a functional layer or a layer to incorporate active ingredients. Several well-known techniques include spraying, dipping, casting, chemical vapor deposition, physical vapor deposition, and screen-printing coating. Spray coating, or deposition, is the term used to describe the process of producing a coating on a thin layer by blasting active substances using a high-pressure nozzle. Dip coating involves immersing the thin film in the coating solution and allowing it to dry. Cast coating, composed of active substances dissolved in a suitable solvent, is applied to the film surface and allowed to dry until the solvent completely disappears. The coating technique is influenced by the adsorbing agents’ and substrate’s physical and chemical makeup [128].

Bruni et al. developed an emulsion electrospinning process with an interior coating for food packaging applications. Soy protein isolates and polyvinyl alcohol (PVA) were used to encapsulate beta-carotene, which was then directly coated onto the polyhydroxybutyrate-co-valerate (PHB92/PHV8) film. In addition, an annealing procedure was used to increase adhesion. The electrospun packing sheet controls the release of the active carotenoid component beta-carotene in soybean oil [119]. Another study looked at the impact of beta-carotene release from nanocapsules integrated into the xanthan gum coating on the physicochemical qualities of fresh-cut melon. The untreated and coated melons were compared. A good link was identified between the release of beta-carotene and xanthan gum treatment, with increased whiteness index (≤10%) and firmness (≤2%). These enhanced the characteristics of the polysaccharide coating matrix and increased its storage life to 21 days at 4 °C [129]. Recent investigations into different carotenoids from various sources and their application and impact on the composites are listed in Table 2.

## 6. Application in Food Packaging Incorporated with Carotenoids

Carotenoids such as β-carotene, β-cryptoxanthin, α-carotene, lutein, β-apo-8-carotenal, astaxanthin, zeaxanthin, and canthaxanthin are the most common natural pigments that are utilized in food packaging applications. These natural pigments are used as food freshness indicators, improving and enhancing the food’s characteristics and properties. The pH value corresponds to the stability of carotenoids and is indicated by a color change. Recent developments in active and intelligent food packaging films incorporating different carotenoid pigments are depicted in Table 3.

**Figure 5:**
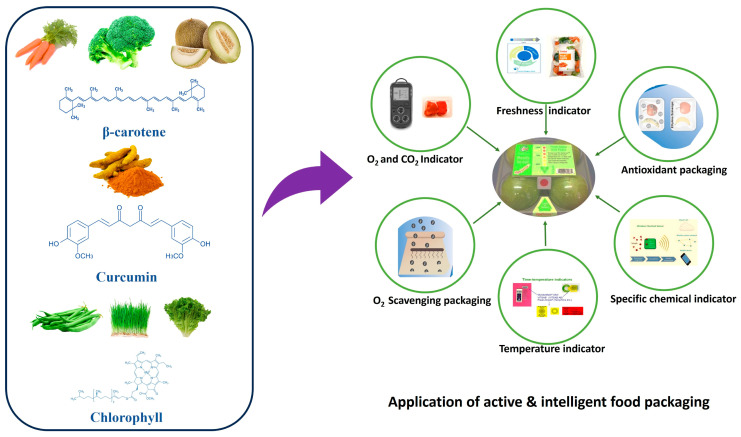
Utilization of different carotenoids in the development of packaging and their application for real-time food storage analysis.

A research study by Medin-Jaramillo developed active and intelligent biodegradable packaging films from cassava starch with different basil and green tea extracts. The results depicted the changes in the color of the basil and tea extracts, which can change the color of the packaging films and act as an indicator of food quality [148]. β-carotene and other carotenoids, such as alpha-tocopherol, have also been explored in food packaging applications. These pigments are applied to foods with high fat content, and absorption is increased by a fatty diet due to the lipophilic nature of carotenoids. Research by Asdagh and Pirsa prepared pectin/nanoclay/carum optimum essential oils/β-carotene-based films and determined the color change in butter. The results showed that the oxidative changes in the butter were detected by the color change from orange to light yellow. A similar study was conducted by Assis et al. by developing a biodegradable cassava starch film incorporating β-carotene nanocapsules [139]. This study found that incorporating the β-carotene nanocapsules enhanced the films’ thermal stability and decreased the oxidation rate in sunflower oil [135]. Various active and intelligent packaging has been developed incorporating different carotenoids, as illustrated in Figure 5.

In a recent work, low-density polyethylene-ethylene vinyl alcohol-polyethylene terephthalate (LDPE/EVOH/PET)-based films containing β-carotene was developed [132]. It was observed that the films with no β-carotene showed an orange color and an increase in the oxidation time. However, with the addition of β-carotene, there was a significant change in the thermal stability and an increase in the oxygen stability of the films. The films’ antioxidative and oxygen-scavenging abilities were evaluated by packaging peanuts in the films for three months at 40 °C, and it was observed that the hexanal content remained constant while being much higher in the controlled films. Tupuna-Yerovi et al. developed food packaging films with sodium alginate as a polymer matrix. They incorporated lycopene and β-carotene, which improved the films’ thermal stability and light transmission. The oxidative effect of the β-carotene and lycopene on the sunflower oil was analyzed, and it was found that the films containing these pigments showed a lower oxidation rate compared to the plastic packaging and control films [107].

Bixin is another natural carotenoid pigment obtained from annatto seeds and contains coloring and antioxidant properties. Stoll et al., in their research work, developed an active polylactic acid-based packaging film with the addition of bixin as a coloring agent. The results indicated that the films showed 90% UV-blocking properties. The antioxidant properties of the films were analyzed on sunflower and other oils, and it was observed that the shelf life of sunflower oil increased with the decrease in the peroxide value [130]. Another researcher developed a biodegradable colored film based on poly(3-hydroxybutyrate) (PHA), adding annatto as a coloring agent. It was observed that the photodegradation of the films under UV-A was enhanced [149]. Nanoencapsulation of bixin has been explored in cassava starch-based biodegradable active packaging-based films by Pagno et al. in their study [144]. The results indicated that the higher concentration of bixin nanocapsules in the films improved the films’ mechanical properties and enhanced their UV-blocking characteristics. The shelf life of the sunflower oil was indicated by the packaging of the developed films, which showed that the films have antioxidant properties by lowering the oxidation rate, which improved the quality of the sunflower oil.

Lycopene is a type of carotenoid pigment that is unsaturated and acyclic and is extracted from tomatoes. Red amaranth has antioxidant and red-colored pigment characteristics. PVA and gelatin-based films were fabricated by incorporating lycopene from tomato extract, and the antimicrobial and antioxidant properties of the films were examined. The results indicated that the films were effective against *Staphylococcus aureus* and *Bacillus cereus.* The shelf life of the chicken meat was observed by packaging it in the films and depicting the antioxidant properties of the films [142]. A similar study by Assis et al. developed a film based on cassava starch with the addition of lycopene nanocapsules. Compared to the control films, the fabricated film with lycopene had higher tensile strength and light barrier properties. Sunflower oil was packed in the fabricated films, which acted as a potential application in preventing oxidation [143]. Cellulose acetate-based packaging films have been fabricated with the addition of norbixin, zeaxanthin, or lycopene, and it was found that the films containing lycopene exhibited higher antioxidant properties as compared to zeaxanthin and norbixin [36].

The various applications of the carotenoids showed that they can be utilized as a colorant, an antioxidant, and an antimicrobial agent, as well as UV blocking in the packaging films, which not only enhances the quality of the food product but also increases the shelf life, thus improving food safety and security. Using carotenoids can be useful for the food packaging industry.

## 7. Safety Aspects and Environmental Impact of Carotenoid-Based Food Packaging

Synthetic colorant can cause a migratory toxic effect on the packed product and the consumers when it goes under high pressure or temperature. Synthetic dye has significant drawbacks, including using hazardous solvents inappropriate for the food business and the toxicity, carcinogenicity, and allergenicity of synthetic dyes instead of natural colorants. Additionally, colorimetric sensor technology is not sophisticated enough for industrial usage and is not readily available. Using natural carotenoids with synthetic colorants has attracted consumers and producers globally. Consumers’ preferences, acceptability, and choices are strongly determined by the sources and methods of production and incorporation techniques associated with developing films containing carotenoids. Smart packaging (active and intelligent systems) based on biopolymers and outfitted with colorimetric indications/sensors, particularly natural colorants (pH indicators), has become increasingly popular among customers worldwide. In addition to safeguarding food from dangers, these intelligent packaging films based on natural colorants also increase food safety and quality, extend product shelf life, preserve food freshness for extended periods, inform consumers about the conditions of the product in real-time, and avoid the environmental issues of conventional packaging and synthetic dyes.

Moreover, utilizing chemically reactive natural colors in active and smart packaging—even when combined with nanoparticles—could be a fruitful strategy that helps the food sector by cutting down on food waste, food-borne illnesses, spoilage, and product degradation. A quick and simple way to keep an eye on the quality, safety, and freshness of packed goods in real-time is to change the film’s color or the packaging. For example, intelligent packaging that displays pH variations in food that has been packaged can serve as a visual pH sensor label. Future developments will also include studying the response of membranes to various factors such as volatile gases and vapors, temperature, etc.; evaluating pigment stability in packaging/film; developing films with improved mechanical properties, including moisture resistance and UV-Vis light blocking; color change analysis through the solution simulation method applied to different foods; and the correlation of pH values with food and sensory deterioration.

## 8. Conclusions

Carotenoids-based natural pigments have excellent potential for developing color-changing active and intelligent packaging indicators. Carotenoids show excellent antioxidant and antimicrobial action. Carotenoids can be easily extracted from natural sources and incorporated into the packaging matrix. The carotenoid-activated packaging film showed good antioxidant activity, a strong antimicrobial effect, excellent pH-responsive color change, and color stability. Carotenoids added to active packaging help improve the packed food’s shelf life by hindering the food-spoiling organism’s growth and limiting the lipid oxidation of food. The intelligent color indicator made using carotenoids can check the freshness of vegetable oils, meat, etc., in real-time. The major drawback with carotenoids and other natural colorants is their color stability; additionally, the solubility of carotenoid-based pigment in fat-soluble solvents also sometimes limits its application. One of the effective strategies to handle this concern is nanoencapsulation, which expands the use of carotenoid pigments in food applications. Even though using these natural pigments in intelligent packaging has good potential, more research is required to enhance the color stability, compatibility with bio-derived polymers, scale-up production of the pigments, effectiveness in different food items, etc. Active and intelligent packaging based on carotenoids is a potential and novel method for improving food packaging safety, quality, and sustainability. This technique uses the inherent qualities of carotenoids, which are strong antioxidants with the unusual ability to change color in response to various environmental stimuli. As the previous discussion shows, adding carotenoids to food packaging materials can have several significant advantages. Above all, carotenoid-based active and intelligent packaging provides an efficient way to improve food shelf life and to monitor and evaluate food quality and freshness in real-time. Reducing the danger of food spoilage, oxidation, and waste, the color changes in response to temperature, oxygen levels, or microbial activity offer a visible and understandable signal for food producers and consumers. With this technology, people can make better decisions about the goods they buy and eat, eventually increasing food sustainability and safety in the market.

## Figures and Tables

**Table 1:** Various carotenoids are biosynthesized using microorganisms.

Microorganism	Isolated Carotenoid and Byproducts	Ref.
Cyanobacteria *Anabaena variabilis*, Aphanizomenon flos-aquae, and Nostoc	canthaxanthin	[59]
*Chlorella pyrenoidosa*	lutein	[60]
*Dictycoccus cinnabarinus*	canthaxanthin	[61]
*Dunaliella salina*	*β*-carotene	[62]
*Dunaliella tertiolecta*	*β*-carotene	[63]
*Haematococcus pluvialis*	astaxanthin	[64]
*Spongiococcum excetricum*	lutein	[61]
Fungi and *Blakeslea trispora* yeasts	*β*-carotene and lycopene	[65]
*Dacrymyces deliquescens*	lutein	[61]
*Phaffia rhodozyma*	astaxanthin and *β*-carotene	[66]
*Rhodotorula glutinis*, and *Rhodotorula graminis*	torulene and *β*-carotene	[67]
*Sporidiobolus salmonicolor*	*β*-carotene	[68]
*Bacterium Bradyrhizobium* sp.	canthaxanthin	[69]
*Mycobacterium lacticola*	astaxanthin	[70]
*Streptomyces chrestomyceticus*	lycopene	[71]
*Rhodococcus maris*	xanthophylls	[72]
*Blakeslea trispora*	*β*-carotene	[73]
*Dunaliella*	*β*-carotene	[74]
*Phaffia rhodozyma*	Astaxanthin	[75]

**Table 2:** Different methods of carotenoid-based composite fabrication and the properties observed.

Carotenoid Source	Polymer Matrix	Fabrication Method	Properties	References
Annatto seeds	Polylactic acid	Melt extrusion	The incorporation of the carotenoids enhanced the UV-blocking properties of UV-A and UV-B by 95% and 75%, respectively.	[130]
Tomatoes Carrots Sunflowers	Sodium alginate	Solution casting	The incorporation of carotenoids reduced the water vapor permeability of the films and improved their thermal stability and light transmission.	[107]
-	Methylcellulose	Solvent casting	The increase in the nanoparticles of carotene showed lower UV transmittance and improved the antioxidant activity of the films.	[131]
-	LDPE/EVOH/PET	Twin extrusion	The incorporation of peanut β-carotene in films delayed oxidative degradation.	[132]
Bixin	Polylactic acid	Solvent casting	The films exhibited higher UV shielding properties, up to 95% of UV-A and 90% of UV-B.	[108]
Carrot	Pectin	Vacuum filtration	Carrot-based pectin improved the film’s mechanical properties and enhanced its antioxidant activity.	[27]
Tomatoes	Polyvinyl alcohol/chitosan	Solvent casting	There was an increase in the antimicrobial activity against *S. aureus* and *P. aeruginosa*, with a MIC of <0.078 mg DW/mL.	[133]
Tomatoes	Polylactic acid/titanium oxide	Solvent casting	The moisture content, antioxidant activity, and film thickness were increased, and higher antimicrobial properties of the films against *S. aureus* and *E. coli* were observed.	[134]
-	Soy protein isolates/polyvinyl alcohol	Electrospinning	The electrospinning coating technique showed higher encapsulation.	[119]
Annatto seeds Tomatoes Goji Berries	Cellulose acetate	Solvent casting	The films exhibited higher antioxidant and barrier properties.	[36]
-	Pectin/nanoclay	Solvent casting	The incorporation of carotene enhances mechanical properties such as flexibility and strength. This also exhibited antioxidant activity of 74.2% and antimicrobial properties against *B. cereus* and *E. coli*.	[135]
Goji berry extract	Sword bean starch	Solution casting	The presence of carotenoids improved the UV-blocking and antioxidant properties by 95.66%.	[136]
*Solanum lycopersicum* L.	Gelatin	Solvent casting	The incorporation of the carotenoid exhibited lower tensile strength but enhanced elongation. The film also showed higher UV-blocking properties.	[137]
Blue carb shells	Chitosan	Solvent casting	The addition of carotene protein exhibited antimicrobial properties of 18 mm against *S. enterica*, 17 mm against *E. coli* and *K. pneumoniae*, and 16 mm against *S. aureus*.	[138]
Carrots	Cassava starch	Solvent casting	The films showed higher antioxidant and light barrier properties and elongation at a rupture with the incorporation of the carotene.	[139]
-	Chitosan/starch nanostructures	Solution casting	The films showed higher antioxidant properties.	[140]
-	Low-density polyethylene	Twin Extrusion	There was a significant reduction in thickness and mechanical, thermal, and water vapor barrier properties.	[141]
Tomato, carrot, and annatto seed	Polylactic acid	Solvent casting	The incorporation of carotenoids exhibited reduced oxygen permeability and high light barrier properties. The film also exhibited a higher elasticity of up to 50%.	[32]
*Solanum lycopersicum*	Polyvinyl alcohol/gelatin	Solution casting	Exhibited lower mechanical properties, higher antimicrobial activity against *S. aureus* and *B. cereus*, and antioxidant properties of 80%.	[142]
Tomatoes	Cassava starch	Solvent casting	Adding lycopene nanocapsules also increased the UV blocking property to 0.01%, as well as the antioxidant activity of the sunflower oil, with a peroxide value of 70.81 ± 0.15 mEq/kg.	[143]
Poly-e-caprolactone	Cassava starch	Solution casting	The films showed higher elongation at break, UV shielding, and antioxidant properties.	[144]
Tomatoes	Shrimp muscle protein	Solution casting	The films showed reduced water solubility to 30% in all the film samples at day 32 and higher UV-blocking properties.	[145]
-	Polyvinyl alcohol/polyethylene oxide	Electrospinning	The electrospinning technique of carotene exhibited UV shielding capability.	[146]
Carrot	Residual carrot/gelatin	Solvent casting	The increasing carotene has also contributed to the greater values of opacity and Young’s modulus, lower light transmission, and elongation at break.	[147]
	White paper	Inkjet printing	The hue of all printed QR code labels is responsive to ammonia solution concentrations, proving that pH-sensitive QR code labels are obtained.	[127]

**Table 3:** Different food packaging applications with the incorporation of carotenoids.

Polymer Matrix	Carotenoid	Food	Food Property Observations	References
Polylactic acid	Bixin	Sunflower oil	The films decreased the light transmission of UV-A by around 0.3% and UV-B by around 1.8%. The films containing bixin shielded oxygen-sensitive food from degradation and decreased the level of peroxides in sunflower oil (below 10 mEq/kg).	[130]
Sodium alginate	β-carotene and lycopene	Sunflower oil	Incorporating lycopene and β-carotene showed a remarkable protective effect on sunflower oil, with a peroxide index of 9.19 ± 1.58 mEq/kg.	[107]
LDPE/EVOH/PET	β-carotene	Peanuts	The peanuts packaged in β-carotene films had delayed oxidative degradation, with an oxygen-absorbing capacity of 1.7 ± 0.3 mL O_2_ per g.	[132]
Polylactic acid	Bixin	Food oil	Incorporating bixin in the films exhibited UV-A transmission values below 10% and UV-B light transmittance below 15%, resulting in lower oxidation properties.	[108]
Carrot pectin	Lipophilic carotenoid	Vegan ripened cashew	The developed film was able to preserve the vegan ripened cashew by improving the antioxidant properties of the film with carotenoids.	[27]
Cellulose acetate	Norbixin, zeaxanthin, or lycopene	Sunflower oil	The films containing norbixin and lycopene exhibited higher antioxidant properties than zeaxanthin and antimicrobial properties with inhibition zone diameters for *B. cereus* and *E. coli.*	[36]
Soy protein isolate/polyvinyl alcohol	β-carotene	Soybean oil	The electrospun coating showed a higher encapsulation of 65.0% ± 2.6%. They were 51.4% ± 0.9% effectively incorporated within their cores. The in vitro release assay in soybean oil that simulates fatty foods showed that the heat treatment (annealing) promoted a slower and more sustained release of the bioactive in the release medium.	[119]
Pectin/nanoclay	β-carotene	Butter	The incorporation of film exhibited a synergistic effect on the antimicrobial activity against the growth of *B. cereus* and *E. coli*. Therefore, antioxidant properties had a peroxide value of 1.1 in 90 days.	[135]
Cassava starch	β-carotene	Sunflower oil	The addition of β-carotene in the films exhibited a lower oxidation rate with an increase in peroxide value during the 30 days of storage, 2.22 ± 0.13 mEq/kg to 274.97 ± 0.45 mEq/kg, and higher UV-transmission properties with a value of 35.47 ± 1.51% at 210 nm.	[139]
Polyvinyl alcohol/gelatin	Lycopene	Chicken meat	The films incorporated with carotenoid enhanced the film’s antimicrobial properties against *S. aureus* and *B. cereus,* which increased the shelf life of chicken meat by preventing spoilage.	[142]
Cassava starch	Lycopene nanocapsules	Sunflower oil	The incorporation of lycopene in the films improved the UV-blocking property as well as increased the antioxidant activity of the sunflower oil.	[143]
Cassava starch	Bixin nanocapsules	Sunflower oil	The incorporation of bixin increased the oxidation rate of the sunflower oil, from an initial peroxide value of 1.92 ± 0.15 mEq/kg to 188.16 ± 3.56 mEq/kg after 30 days of storage.	[144]

## Data Availability

Data is contained within the article.

## References

[1] He X., Pu Y., Chen L., Jiang H., Xu Y., Cao J., Jiang W. (2023). A Comprehensive Review of Intelligent Packaging for Fruits and Vegetables: Target Responders, Classification, Applications, and Future Challenges. Compr. Rev. Food Sci. Food Saf..

[2] Mohammadian E., Alizadeh-Sani M., Jafari S.M. (2020). Smart Monitoring of Gas/Temperature Changes within Food Packaging Based on Natural Colorants. Compr. Rev. Food Sci. Food Saf..

[3] Roy S., Priyadarshi R., Ezati P., Rhim J.W. (2022). Curcumin and Its Uses in Active and Smart Food Packaging Applications—A Comprehensive Review. Food Chem..

[4] Soltani Firouz M., Mohi-Alden K., Omid M. (2021). A Critical Review on Intelligent and Active Packaging in the Food Industry: Research and Development. Food Res. Int..

[5] Martinez M.M., Versino F., Ortega F., Monroy Y., Rivero S., López O.V., García M.A. (2023). Sustainable and Bio-Based Food Packaging: A Review on Past and Current Design Innovations. Foods.

[6] Roy S., Rhim J.-W. (2020). Anthocyanin Food Colorant and Its Application in PH-Responsive Color Change Indicator Films. Crit. Rev. Food Sci. Nutr..

[7] Deshmukh R.K., Hakim L., Akhila K., Ramakanth D., Gaikwad K.K. (2023). Nano Clays and Its Composites for Food Packaging Applications. Int. Nano Lett..

[8] Roy S., Zhang W., Biswas D., Ramakrishnan R., Rhim J.-W. (2023). Grapefruit seed extract-added functional films and coating for active packaging applications: A review. Molecules.

[9] Nemes S.A., Szabo K., Vodnar D.C. (2020). Applicability of Agro-Industrial By-Products in Intelligent Food Packaging. Coatings.

[10] Kumar J., Akhila K., Kumar P., Deshmukh R.K., Gaikwad K.K. (2023). Novel Temperature-Sensitive Label Based on Thermochromic Ink for Hot Food Packaging and Serving Applications. J. Therm. Anal. Calorim..

[11] Balbinot-Alfaro E., Craveiro D.V., Lima K.O., Costa H.L.G., Lopes D.R., Prentice C. (2019). Intelligent Packaging with PH Indicator Potential. Food Eng. Rev..

[12] Yousefi H., Su H.M., Imani S.M., Alkhaldi K., Filipe C.D., Didar T.F. (2019). Intelligent Food Packaging: A Review of Smart Sensing Technologies for Monitoring Food Quality. ACS Sens..

[13] Kumar L., Deshmukh R.K., Hakim L., Gaikwad K.K. (2023). Halloysite Nanotube as a Functional Material for Active Food Packaging Application: A Review. Food Bioprocess Technol..

[14] Grzebieniarz W., Biswas D., Roy S., Jamróz E. (2023). Advances in biopolymer-based multi-layer film preparations and food packaging applications. Food Packag. Shelf Life.

[15] Deshmukh R.K., Akhila K., Ramakanth D., Gaikwad K.K. (2022). Guar Gum/Carboxymethyl Cellulose Based Antioxidant Film Incorporated with Halloysite Nanotubes and Litchi Shell Waste Extract for Active Packaging. Int. J. Biol. Macromol..

[16] Rout S., Tambe S., Deshmukh R.K., Mali S., Cruz J., Srivastav P.P., Amin P.D., Gaikwad K.K., de Aguiar Andrade E.H., de Oliveira M.S. (2022). Recent Trends in the Application of Essential Oils: The next Generation of Food Preservation and Food Packaging. Trends Food Sci. Technol..

[17] Niponsak A., Laohakunjit N., Kerdchoechuen O., Wongsawadee P. (2016). Development of Smart Colourimetric Starch–Based Indicator for Liberated Volatiles during Durian Ripeness. Food Res. Int..

[18] Ran R., Wang L., Su Y., He S., He B., Li C., Wang C., Liu Y., Chen S. (2021). Preparation of PH-Indicator Films Based on Soy Protein Isolate/Bromothymol Blue and Methyl Red for Monitoring Fresh-Cut Apple Freshness. J. Food Sci..

[19] Amjadi S., Nazari M., Alizadeh S.A., Hamishehkar H. (2020). Multifunctional Betanin Nanoliposomes-Incorporated Gelatin/Chitosan Nanofiber/ZnO Nanoparticles Nanocomposite Film for Fresh Beef Preservation. Meat Sci..

[20] de Oliveira Filho J.G., de Sousa T.L., Bertolo M.R.V., Bogusz Junior S., Mattoso L.H.C., Pimentel T.C., Egea M.B. (2023). Next-Generation Food Packaging: Edible Bioactive Films with Alginate, Mangaba Pulp (*Hancornia speciosa*), and *Saccharomyces boulardii*. Food Biosci..

[21] Ahmed M., Bose I., Goksen G., Roy S. (2023). Himalayan Sources of Anthocyanins and Its Multifunctional Applications: A Review. Foods.

[22] Roy S., Chawla R., Santhosh R., Thakur R., Sarkar P., Zhang W. (2023). Agar-based edible films and food packaging application: A comprehensive review. Trends Food Sci. Technol..

[23] Idrovo Encalada A.M., De’Nobili M.D., Ponce A.N.M., Stortz C.A., Fissore E.N., Rojas A.M. (2021). Antioxidant Edible Film Based on a Carrot Pectin-Enriched Fraction as an Active Packaging of a Vegan Cashew Ripened Cheese. Int. J. Food Sci. Technol..

[24] Sigurdson G.T., Tang P., Giusti M.M. (2017). Natural Colorants: Food Colorants from Natural Sources. Annu. Rev. Food Sci. Technol..

[25] Rennan I., Vieira S., Paula A., De De Carvalho A., Conte-Junior C.A. (2022). Recent Advances in Biobased and Biodegradable Polymer Nanocomposites, Nanoparticles, and Natural Antioxidants for Antibacterial and Antioxidant Food Packaging Applications. Compr. Rev. Food Sci. Food Saf..

[26] Meléndez-Martínez A.J., Mandić A.I., Bantis F., Böhm V., Borge G.I.A., Brnčić M., Bysted A., Cano M.P., Dias M.G., Elgersma A. (2021). A Comprehensive Review on Carotenoids in Foods and Feeds: Status Quo, Applications, Patents, and Research Needs. Crit. Rev. Food Sci. Nutr..

[27] Ashokkumar V., Flora G., Sevanan M., Sripriya R., Chen W.H., Park J.H., Rajesh banu J., Kumar G. (2023). Technological Advances in the Production of Carotenoids and Their Applications—A Critical Review. Bioresour. Technol..

[28] Deshmukh R.K., Hakim L., Gaikwad K.K. (2023). Active Packaging Materials. Curr. Food Sci. Technol. Rep..

[29] Bhatt T., Patel K. (2020). Carotenoids: Potent to Prevent Diseases Review. Nat. Prod. Bioprospect.

[30] Assis R.Q., Pagno C.H., Stoll L., Rios P.D.A., de Oliveira Rios A., Olivera F.C. (2021). Active Food Packaging of Cellulose Acetate: Storage Stability, Protective Effect on Oxidation of Riboflavin and Release in Food Simulants. Food Chem..

[31] Anushikha, Deshmukh R.K., Kunam P.K., Gaikwad K.K. (2023). Guar Gum Based Flexible Packaging Material with an Active Surface Reinforced by Litchi Shell Derived Micro Fibrillated Cellulose and Halloysite Nanotubes. Sustain. Chem. Pharm..

[32] Stoll L., Rech R., Flôres S.H., Nachtigall S.M.B., de Oliveira Rios A. (2018). Carotenoids Extracts as Natural Colorants in Poly(Lactic Acid) Films. J. Appl. Polym. Sci..

[33] Cassani L., Marcovich N.E., Gomez-Zavaglia A. (2022). Valorization of Fruit and Vegetables Agro-Wastes for the Sustainable Production of Carotenoid-Based Colorants with Enhanced Bioavailability. Food Res. Int..

[34] Stoll L., Rech R., Flôres S.H., Nachtigall S.M.B., de Oliveira Rios A. (2019). Poly(Acid Lactic) Films with Carotenoids Extracts: Release Study and Effect on Sunflower Oil Preservation. Food Chem..

[35] Pirsa S., Asadi S. (2021). Innovative Smart and Biodegradable Packaging for Margarine Based on a Nano Composite Polylactic Acid/Lycopene Film. Food Addit. Contam. Part A.

[36] Assis R.Q., Rios P.D.A., de Oliveira Rios A., Olivera F.C. (2020). Biodegradable Packaging of Cellulose Acetate Incorporated with Norbixin, Lycopene or Zeaxanthin. Ind. Crops Prod..

[37] Jain P., Tripathi S., Deshmukh R.K., Gaikwad K.K., Singh S. (2023). Functionalization of Sugarcane Bagasse–Based Paper with Amla Pomace/Titanium Dioxide Nanoparticles Providing Antimicrobial Protection for Food Safety. Biomass Convers. Biorefinery.

[38] de Oliveira Filho J.G., Bertolo M.R.V., Fernandes S.S., Lemes A.C., da Cruz Silva G., Junior S.B., de Azeredo H.M.C., Mattoso L.H.C., Egea M.B. (2024). Intelligent and Active Biodegradable Biopolymeric Films Containing Carotenoids. Food Chem..

[39] Ghosh S., Roy S., Naskar J., Kole R.K. (2023). Plant-Mediated Synthesis of Mono-and Bimetallic (Au–Ag) Nanoparticles: Future Prospects for Food Quality and Safety. J. Nanomater..

[40] Oun A.A., Roy S., Shin G.H., Yoo S., Kim J.T. (2023). pH-sensitive smart indicators based on cellulose and different natural pigments for tracing kimchi ripening stages. Int. J. Biol. Macromol..

[41] Gaikwad K.K., Deshmukh R.K., Lee Y.S. (2022). Natural Phenolic Compound Coated Oxygen Scavenging Active Polyolefin Film for Preserving Quality of Fish Cake. Biomass Convers. Biorefinery.

[42] Tripathi S., Kumar L., Deshmukh R.K., Gaikwad K.K. (2023). Ultraviolet Blocking Films for Food Packaging Applications. Food Bioprocesss Technol..

[43] Deshmukh R.K., Ramakanth D., Akhila K., Gaikwad K.K. (2023). Natural Clay-Based Food Packaging Films. Natural Materials for Food Packaging Application.

[44] Rodriguez-Concepcion M., Avalos J., Bonet M.L., Boronat A., Gomez-Gomez L., Hornero-Mendez D., Limon M.C., Meléndez-Martínez A.J., Olmedilla-Alonso B., Palou A. (2018). A Global Perspective on Carotenoids: Metabolism, Biotechnology, and Benefits for Nutrition and Health. Prog. Lipid Res..

[45] Bell J.G., McEvoy J., Tocher D.R., Sargent J.R. (2000). Depletion of α-Tocopherol and Astaxanthin in Atlantic Salmon (Salmo Salar) Affects Autoxidative Defense and Fatty Acid Metabolism. J. Nutr..

[46] Botella-Pavía P., Rodríguez-Concepción M. (2006). Carotenoid Biotechnology in Plants for Nutritionally Improved Foods. Physiol. Plant..

[47] Papapostolou H., Kachrimanidou V., Alexandri M., Plessas S., Papadaki A., Kopsahelis N. (2023). Natural Carotenoids: Recent Advances on Separation from Microbial Biomass and Methods of Analysis. Antioxidants.

[48] Dias M.G., Olmedilla-Alonso B., Hornero-Méndez D., Mercadante A.Z., Osorio C., Vargas-Murga L., Meléndez-Martínez A.J. (2018). Comprehensive Database of Carotenoid Contents in Ibero-American Foods. A Valuable Tool in the Context of Functional Foods and the Establishment of Recommended Intakes of Bioactives. J. Agric. Food Chem..

[49] Zhou W., Niu Y., Ding X., Zhao S., Li Y., Fan G., Zhang S., Liao K. (2020). Analysis of Carotenoid Content and Diversity in Apricots (*Prunus armeniaca* L.) Grown in China. Food Chem..

[50] Chisté R.C., Mercadante A.Z. (2012). Identification and Quantification, by HPLC-DAD-MS/MS, of Carotenoids and Phenolic Compounds from the Amazonian Fruit Caryocar Villosum. J. Agric. Food Chem..

[51] Turkiewicz I.P., Wojdyło A., Tkacz K., Nowicka P. (2020). Carotenoids, Chlorophylls, Vitamin E and Amino Acid Profile in Fruits of Nineteen Chaenomeles Cultivars. J. Food Compos. Anal..

[52] Delgado-Pelayo R., Hornero-Méndez D. (2012). Identification and Quantitative Analysis of Carotenoids and Their Esters from Sarsaparilla (*Smilax aspera* L.) Berries. J. Agric. Food Chem..

[53] Riedl K.M., Choksi K., Wyzgoski F.J., Scheerens J.C., Schwartz S.J., Reese R.N. (2013). Variation in Lycopene and Lycopenoates, Antioxidant Capacity, and Fruit Quality of Buffaloberry (*Shepherdia Argentea* [Pursh]Nutt.). J. Food Sci..

[54] Rodríguez-Bernaldo de Quirós A., Costa H.S. (2006). Analysis of Carotenoids in Vegetable and Plasma Samples: A Review. J. Food Compos. Anal..

[55] Sugiura M., Nakamura M., Ikoma Y., Yano M., Ogawa K., Matsumoto H., Kato M., Ohshima M., Nagao A. (2006). Serum Carotenoid Concentrations Are Inversely Associated with Serum Aminotransferases in Hyperglycemic Subjects. Diabetes Res. Clin. Pract..

[56] Deshmukh R.K., Gaikwad K.K. (2022). Natural Antimicrobial and Antioxidant Compounds for Active Food Packaging Applications. Biomass Convers. Biorefinery.

[57] Mezzomo N., Ferreira S.R.S. (2016). Carotenoids Functionality, Sources, and Processing by Supercritical Technology: A Review. J. Chem..

[58] Saini R.K., Keum Y.-S. (2018). Carotenoid Extraction Methods: A Review of Recent Developments. Food Chem..

[59] Krajewska M., Szymczak-Żyła M., Kobos J., Witak M., Kowalewska G. (2019). Canthaxanthin in Recent Sediments as an Indicator of Heterocystous Cyanobacteria in Coastal Waters. Oceanologia.

[60] Sampathkumar S.J., Gothandam K.M. (2019). Sodium Bicarbonate Augmentation Enhances Lutein Biosynthesis in Green Microalgae Chlorella Pyrenoidosa. Biocatal. Agric. Biotechnol..

[61] Schroeder W.A., Johnson E.A. (1995). Singlet Oxygen and Peroxyl Radicals Regulate Carotenoid Biosynthesis in *Phaffia rhodozyma*. J. Biol. Chem..

[62] Xi Y., Kong F., Chi Z. (2021). ROS Induce β-Carotene Biosynthesis Caused by Changes of Photosynthesis Efficiency and Energy Metabolism in *Dunaliella salina* Under Stress Conditions. Front. Bioeng. Biotechnol..

[63] Zarandi-Miandoab L., Hejazi M.-A., Bagherieh-Najjar M.-B., Chaparzadeh N. (2019). Optimization of the Four Most Effective Factors on β-Carotene Production by *Dunaliella salina* Using Response Surface Methodology. Iran. J. Pharm. Res..

[64] de Moraes L.B.S., Mota G.C.P., dos Santos E.P., Campos C.V.F.d.S., da Silva B.A.B., Olivera Gálvez A., de Souza Bezerra R. (2023). Haematococcus Pluvialis Cultivation and Astaxanthin Production Using Different Nitrogen Sources with Pulse Feeding Strategy. Biomass Convers. Biorefinery.

[65] Sandmann G. (2022). Carotenoids and Their Biosynthesis in Fungi. Molecules.

[66] Liu Y.S., Wu J.Y. (2006). Use of N-Hexadecane as an Oxygen Vector to Improve *Phaffia rhodozyma* Growth and Carotenoid Production in Shake-Flask Cultures. J. Appl. Microbiol..

[67] Igreja W.S., de Maia F.A., Lopes A.S., Chisté R.C. (2021). Biotechnological Production of Carotenoids Using Low Cost-Substrates Is Influenced by Cultivation Parameters: A Review. Int. J. Mol. Sci..

[68] Valduga E., Valério A., Treichel H., Furigo Júnior A., Di Luccio M. (2009). Optimization of the Production of Total Carotenoids by Sporidiobolus Salmonicolor (CBS 2636) Using Response Surface Technique. Food Bioprocess Technol..

[69] Hannibal L., Lorquin J., D’Ortoli N.A., Garcia N., Chaintreuil C., Masson-Boivin C., Dreyfus B., Giraud E. (2000). Isolation and Characterization of Canthaxanthin Biosynthesis Genes from the Photosynthetic Bacterium *Bradyrhizobium* Sp. Strain ORS278. J. Bacteriol..

[70] Karpiński T.M., Ożarowski M., Alam R., Łochyńska M., Stasiewicz M. (2021). What Do We Know about Antimicrobial Activity of Astaxanthin and Fucoxanthin?. Mar. Drugs.

[71] Di Salvo E., Lo Vecchio G., De Pasquale R., De Maria L., Tardugno R., Vadalà R., Cicero N. (2023). Natural Pigments Production and Their Application in Food, Health and Other Industries. Nutrients.

[72] Cappelletti M., Presentato A., Piacenza E., Firrincieli A., Turner R.J., Zannoni D. (2020). Biotechnology of Rhodococcus for the Production of Valuable Compounds. Appl. Microbiol. Biotechnol..

[73] Papadaki E., Mantzouridou F.T. (2021). Natural β-Carotene Production by *Blakeslea trispora* Cultivated in Spanish-Style Green Olive Processing Wastewaters. Foods.

[74] Wolf L., Cummings T., Müller K., Reppke M., Volkmar M., Weuster-Botz D. (2021). Production of Β-carotene with *Dunaliella Salina* CCAP19/18 at Physically Simulated Outdoor Conditions. Eng. Life Sci..

[75] Luna-Flores C.H., Wang A., von Hellens J., Speight R.E. (2022). Towards Commercial Levels of Astaxanthin Production in *Phaffia rhodozyma*. J. Biotechnol..

[76] Young A., Lowe G. (2018). Carotenoids—Antioxidant Properties. Antioxidants.

[77] Srivastava R. (2021). Physicochemical, Antioxidant Properties of Carotenoids and Its Optoelectronic and Interaction Studies with Chlorophyll Pigments. Sci. Rep..

[78] Squillaci G., Parrella R., Carbone V., Minasi P., La Cara F., Morana A. (2017). Carotenoids from the Extreme Halophilic Archaeon *Haloterrigena turkmenica*: Identification and Antioxidant Activity. Extremophiles.

[79] Gao Y., Ligia Focsan A., Kispert L.D. (2020). The Effect of Polarity of Environment on the Antioxidant Activity of Carotenoids. Chem. Phys. Lett..

[80] Merhan O. (2017). The Biochemistry and Antioxidant Properties of Carotenoids. Carotenoids.

[81] Zhao Y., Guo L., Xia Y., Zhuang X., Chu W. (2019). Isolation, Identification of Carotenoid-Producing Rhodotorula Sp. from Marine Environment and Optimization for Carotenoid Production. Mar. Drugs.

[82] Yoo A.Y., Alnaeeli M., Park J.K. (2016). Production Control and Characterization of Antibacterial Carotenoids from the Yeast *Rhodotorula mucilaginosa* AY-01. Process Biochem..

[83] Dumitriu C., Ungureanu C., Popescu S., Tofan V., Popescu M., Pirvu C. (2015). Ti Surface Modification with a Natural Antioxidant and Antimicrobial Agent. Surf. Coat. Technol..

[84] Ungureanu C., Ferdes M. (2012). Evaluation of Antioxidant and Antimicrobial Activities of Torularhodin. Adv. Sci. Lett..

[85] Kusmita L., Tatsa Y.A., Franyoto Y.D., Sabdono A., Trianto A., Radjasa O.K. (2021). Antibacterial Activity of Carotenoid from Bacterial Symbiont Virgibacillus Salarius Strain 19. PP. Sc. 1.6 against MDR E. Coli and MRSA. Egypt. J. Aquat. Biol. Fish..

[86] Abou-Dobara M.I. (2013). Antibacterial Activity of Some Marine Algal Extracts against Most Nosocomial Bacterial Infections. Egypt. J. Bot..

[87] Karpiński T.M., Adamczak A. (2019). Fucoxanthin—An Antibacterial Carotenoid. Antioxidants.

[88] Bose I., Nousheen, Roy S., Yaduvanshi P., Sharma S., Chandel V., Biswas D. (2023). Unveiling the potential of marine biopolymers: Sources, classification, and diverse food applications. Materials.

[89] Liu J., Yong H., Liu Y., Qin Y., Kan J., Liu J. (2019). Preparation and Characterization of Active and Intelligent Films Based on Fish Gelatin and Haskap Berries (*Lonicera Caerulea* L.) Extract. Food Packag. Shelf Life.

[90] Jaber B.A., Majeed K.R. (2021). Antioxidant and Antibacterial Activity of Β-Carotene Pigment Extracted From Parococcus Homiensis Strain BKA7 Isolated from Air of Basra, Iraq. Ann. Rom. Soc. Cell. Biol..

[91] Gutiérrez-del-Río I., Fernández J., Lombó F. (2018). Plant Nutraceuticals as Antimicrobial Agents in Food Preservation: Terpenoids, Polyphenols and Thiols. Int. J. Antimicrob. Agents.

[92] Shanmugapriya K., Kim H., Saravana P.S., Chun B.-S., Kang H.W. (2018). Astaxanthin-Alpha Tocopherol Nanoemulsion Formulation by Emulsification Methods: Investigation on Anticancer, Wound Healing, and Antibacterial Effects. Colloids Surf. B. Biointerfaces.

[93] Contreras-Ortiz J.M.E., Barbabosa-Pliego A., Oros-Pantoja R., Aparicio-Burgos J.E., Zepeda-Escobar J.A., Hassan-Moustafa W.H., Ochoa-García L., Alonso-Fresan M.U., Borroto E.T., Vázquez-Chagoyán J.C. (2017). Effects of Astaxanthin in Mice Acutely Infected with Trypanosoma Cruzi. Parasite.

[94] Brendler T., Williamson E.M. (2019). Astaxanthin: How Much Is Too Much? A Safety Review. Phytother. Res..

[95] Edwards J.A., Bellion P., Beilstein P., Rümbeli R., Schierle J. (2016). Review of Genotoxicity and Rat Carcinogenicity Investigations with Astaxanthin. Regul. Toxicol. Pharmacol..

[96] Kalpana S., Priyadarshini S.R., Maria Leena M., Moses J.A., Anandharamakrishnan C. (2019). Intelligent Packaging: Trends and Applications in Food Systems. Trends Food Sci. Technol..

[97] Neves D., Andrade P.B., Videira R.A., de Freitas V., Cruz L. (2022). Berry Anthocyanin-Based Films in Smart Food Packaging: A Mini-Review. Food Hydrocoll..

[98] Nogueira G.F., de Oliveira R.A., Velasco J.I., Fakhouri F.M. (2020). Methods of Incorporating Plant-Derived Bioactive Compounds into Films Made with Agro-Based Polymers for Application as Food Packaging: A Brief Review. Polymers.

[99] Chen P., Xie F., McNally T. (2021). Understanding the Effects of Montmorillonite and Sepiolite on the Properties of Solution-Cast Chitosan and Chitosan/Silk Peptide Composite Films. Polym. Int..

[100] Adnan M., Obyedul Kalam Azad M., Seok Ju H., Min Son J., Ho Park C., Hwan Shin M., Alle M., Ha Cho D. (2020). Development of Biopolymer-Mediated Nanocomposites Using Hot-Melt Extrusion to Enhance the Bio-Accessibility and Antioxidant Capacity of Kenaf Seed Flour. Appl. Nanosci..

[101] Aina K.S., Oladimeji A.O., Agboola F.Z., Oguntayo D.O. (2022). Dimensional Stability and Mechanical Properties of Extruded-Compression Biopolymer Composites Made from Selected Nigerian Grown Wood Species at Varying Proportions. Sci. Rep..

[102] Madruga L.Y.C., Kipper M.J. (2022). Expanding the Repertoire of Electrospinning: New and Emerging Biopolymers, Techniques, and Applications. Adv. Healthc. Mater..

[103] Ren L., Chen J., Lu Q., Han J., Wu H. (2021). Anti-Biofouling Nanofiltration Membrane Constructed by in-Situ Photo-Grafting Bactericidal and Hydrophilic Polymers. J. Memb. Sci..

[104] Li N., Qiao D., Zhao S., Lin Q., Zhang B., Xie F. (2021). 3D Printing to Innovate Biopolymer Materials for Demanding Applications: A Review. Mater. Today Chem..

[105] Gonçalves A., Estevinho B.N., Rocha F. (2019). Characterization of Biopolymer-Based Systems Obtained by Spray-Drying for Retinoic Acid Controlled Delivery. Powder Technol..

[106] Mathew A.P., Oksman K. (2014). Processing of Bionanocomposites: Solution Casting. Handbook of Green Materials: 2 Bionanocomposites: Processing, Characterization And Properties.

[107] Tupuna-Yerovi D.S., Schmidt H., de Oliveira Rios A. (2023). Biodegradable Sodium Alginate Films Incorporated with Lycopene and β-Carotene for Food Packaging Purposes. Food Sci. Technol. Int..

[108] Stoll L., Domenek S., Hickmann Flôres S., Nachtigall S.M.B., de Oliveira Rios A. (2021). Polylactide Films Produced with Bixin and Acetyl Tributyl Citrate: Functional Properties for Active Packaging. J. Appl. Polym. Sci..

[109] Prabha K., Ghosh P., S A., Joseph R.M., Krishnan R., Rana S.S., Pradhan R.C. (2021). Recent Development, Challenges, and Prospects of Extrusion Technology. Future Foods.

[110] Sikora J., Majewski Ł., Puszka A. (2020). Modern Biodegradable Plastics—Processing and Properties: Part I. Materials.

[111] Kim S.M., Roy S., Yoon K.S., Rhim J.-W. (2021). Preparation of low-density polyethylene-and poly (lactide)/poly (butylene adipate-co-terephthalate)-based antibacterial films integrated with elemental sulfur and sulfur nanoparticles. Packag. Technol. Sci..

[112] Leal I.L., Silva Rosa Y.C., Silva Penha J., Cruz Correia P.R., Silva Melo P., Guimarães D.H., Barbosa J.D.V., Druzian J.I., Machado B.A.S. (2019). Development and Application Starch Films: PBAT with Additives for Evaluating the Shelf Life of Tommy Atkins Mango in the Fresh-cut State. J. Appl. Polym. Sci..

[113] Dhar P., Gaur S.S., Soundararajan N., Gupta A., Bhasney S.M., Milli M., Kumar A., Katiyar V. (2017). Reactive Extrusion of Polylactic Acid/Cellulose Nanocrystal Films for Food Packaging Applications: Influence of Filler Type on Thermomechanical, Rheological, and Barrier Properties. Ind. Eng. Chem. Res..

[114] Aman Mohammadi M., Hosseini S.M., Yousefi M. (2020). Application of Electrospinning Technique in Development of Intelligent Food Packaging: A Short Review of Recent Trends. Food Sci. Nutr..

[115] Cheng J., Jun Y., Qin J., Lee S.-H. (2017). Electrospinning versus Microfluidic Spinning of Functional Fibers for Biomedical Applications. Biomaterials.

[116] Rezaei A., Nasirpour A., Fathi M. (2015). Application of Cellulosic Nanofibers in Food Science Using Electrospinning and Its Potential Risk. Compr. Rev. Food Sci. Food Saf..

[117] Sameen D.E., Ahmed S., Lu R., Li R., Dai J., Qin W., Zhang Q., Li S., Liu Y. (2022). Electrospun Nanofibers Food Packaging: Trends and Applications in Food Systems. Crit. Rev. Food Sci. Nutr..

[118] Nieuwland M., Geerdink P., Brier P., van den Eijnden P., Henket J.T.M.M., Langelaan M.L.P., Stroeks N., van Deventer H.C., Martin A.H. (2013). Food-Grade Electrospinning of Proteins. Innov. Food Sci. Emerg. Technol..

[119] Pinheiro Bruni G., de Oliveira J.P., Gómez-Mascaraque L.G., Fabra M.J., Guimarães Martins V., Zavareze E.d.R., López-Rubio A. (2020). Electrospun β-Carotene–Loaded SPI:PVA Fiber Mats Produced by Emulsion-Electrospinning as Bioactive Coatings for Food Packaging. Food Packag. Shelf Life.

[120] Yildiz Z.I., Topuz F., Kilic M.E., Durgun E., Uyar T. (2023). Encapsulation of Antioxidant Beta-Carotene by Cyclodextrin Complex Electrospun Nanofibers: Solubilization and Stabilization of Beta-Carotene by Cyclodextrins. Food Chem..

[121] Kumar D., Gihar S., Shrivash M.K., Kumar P., Kundu P.P. (2020). A Review on the Synthesis of Graft Copolymers of Chitosan and Their Potential Applications. Int. J. Biol. Macromol..

[122] Sadeghi K., Jee H.-W., Paeng K.-J., Seo J. (2021). Photografting of Conducting Polymer onto Polymeric Substrate as Non-Migratory Antioxidant Packaging. React. Funct. Polym..

[123] Sadeghi K., Seo J. (2021). Photografting of Biochelator onto Polypropylene Film as an Antioxidant Clean Label. Food Chem..

[124] Zhang Y., Lim L.-T. (2016). Inkjet-Printed CO_2_ Colorimetric Indicators. Talanta.

[125] Bao F., Liang Z., Deng J., Lin Q., Li W., Peng Q., Fang Y. (2022). Toward Intelligent Food Packaging of Biosensor and Film Substrate for Monitoring Foodborne Microorganisms: A Review of Recent Advancements. Crit. Rev. Food Sci. Nutr..

[126] Luo X., Ho I., Brankovan S., Lim L.-T. (2021). Inkjet-Printed Gradient Colorimetric Indicators for Monitoring Fish Freshness. Food Packag. Shelf Life.

[127] Xu Y., Liu Z., Liu R., Luo M., Wang Q., Cao L., Ye S. (2021). Inkjet-Printed PH-Sensitive QR Code Labels for Real-Time Food Freshness Monitoring. J. Mater. Sci..

[128] Fu Y., Dudley E.G. (2021). Antimicrobial-coated Films as Food Packaging: A Review. Compr. Rev. Food Sci. Food Saf..

[129] Zambrano-Zaragoza M.L., Quintanar-Guerrero D., Del Real A., Piñon-Segundo E., Zambrano-Zaragoza J.F. (2017). The Release Kinetics of β-Carotene Nanocapsules/Xanthan Gum Coating and Quality Changes in Fresh-Cut Melon (Cantaloupe). Carbohydr. Polym..

[130] Stoll L., Maillard M.N., Le Roux E., Hickmann Flôres S., Nachtigall S.M.B., Rios A., Domenek S. (2023). Bixin, a Performing Natural Antioxidant in Active Food Packaging for the Protection of Oxidation Sensitive Food. LWT.

[131] Lino R.C., de Carvalho S.M., Noronha C.M., Sganzerla W.G., da Rosa C.G., Nunes M.R., D’Avila R.F., Zambiazi R.C., Barreto P.L.M. (2022). Production of Methylcellulose Films Functionalized with Poly-ε-Caprolactone Nanocapsules Entrapped β-Carotene for Food Packaging Application. Food Res. Int..

[132] Juan-Polo A., Maestre Pérez S.E., Monedero Prieto M., Sánchez Reig C., Tone A.M., Herranz Solana N., Beltrán Sanahuja A. (2022). Oxygen scavenger and antioxidant LDPE/EVOH/PET-based films containing β-carotene intended for fried peanuts (*Arachis hypogaea* L.) packaging: Pilot scale processing and validation studies. Polymers.

[133] Szabo K., Teleky B.E., Mitrea L., Călinoiu L.F., Martău G.A., Simon E., Varvara R.A., Vodnar D.C. (2020). Active Packaging-Poly (Vinyl Alcohol) Films Enriched with Tomato by-Products Extract. Coatings.

[134] Asadi S., Pirsa S. (2020). Production of Biodegradable Film Based on Polylactic Acid, Modified with Lycopene Pigment and TiO2 and Studying Its Physicochemical Properties. J. Polym. Environ..

[135] Asdagh A., Pirsa S. (2020). Bacterial and Oxidative Control of Local Butter with Smart/Active Film Based on Pectin/Nanoclay/Carum Copticum Essential Oils/β-Carotene. Int. J. Biol. Macromol..

[136] Kim S., Kang J.H., Song K. (2020). Bin Development of a Sword Bean (*Canavalia gladiata*) Starch Film Containing Goji Berry Extract. Food Bioprocess Technol..

[137] López-Palestina C.U., Aguirre-Mancilla C.L., Raya-Pérez J.C., Ramirez-Pimentel J.G., Vargas-Torres A., Hernández-Fuentes A.D. (2019). Physicochemical and Antioxidant Properties of Gelatin-Based Films Containing Oily Tomato Extract (*Solanum Lycopersicum* L.). CYTA—J. Food.

[138] Hamdi M., Nasri R., Li S., Nasri M. (2019). Bioactive Composite Films with Chitosan and Carotenoproteins Extract from Blue Crab Shells: Biological Potential and Structural, Thermal, and Mechanical Characterization. Food Hydrocoll..

[139] Assis R.Q., Pagno C.H., Costa T.M.H., Flôres S.H., de Oliveira Rios A. (2018). Synthesis of Biodegradable Films Based on Cassava Starch Containing Free and Nanoencapsulated β-Carotene. Packag. Technol. Sci..

[140] Hari N., Francis S., Rajendran Nair A.G., Nair A.J. (2018). Synthesis, Characterization and Biological Evaluation of Chitosan Film Incorporated with β-Carotene Loaded Starch Nanocrystals. Food Packag. Shelf Life.

[141] Moura L.E., de Souza C.O., de Oliveira E.A.S., Lemos P.V.F., Druzian J.I. (2018). Bioactive Efficacy of Low-Density Polyethylene Films with Natural Additives. J. Appl. Polym. Sci..

[142] Kanatt S.R., Jethwa T., Sawant K., Chawla S.P. (2017). PVA-Gelatin Films Incorporated with Tomato Pulp: A Potential Primary Food Packaging Film. Int. J. Curr. Microbiol. Appl. Sci..

[143] Assis R.Q., Lopes S.M., Costa T.M.H., Flôres S.H., de Oliveira Rios A. (2017). Active Biodegradable Cassava Starch Films Incorporated Lycopene Nanocapsules. Ind. Crops Prod..

[144] Pagno C.H., de Farias Y.B., Costa T.M.H., de Oliveira Rios A., Flôres S.H. (2016). Synthesis of Biodegradable Films with Antioxidant Properties Based on Cassava Starch Containing Bixin Nanocapsules. J. Food Sci. Technol..

[145] Gómez-Estaca J., Calvo M.M., Sánchez-Faure A., Montero P., Gómez-Guillén M.C. (2015). Development, Properties, and Stability of Antioxidant Shrimp Muscle Protein Films Incorporating Carotenoid-Containing Extracts from Food by-Products. LWT.

[146] de Freitas Zômpero R.H., López-Rubio A., de Pinho S.C., Lagaron J.M., de la Torre L.G. (2015). Hybrid Encapsulation Structures Based on β-Carotene-Loaded Nanoliposomes within Electrospun Fibers. Colloids Surf. B. Biointerfaces.

[147] Iahnke A.O.e.S., Costa T.M.H., de Oliveira Rios A., Flôres S.H. (2015). Residues of Minimally Processed Carrot and Gelatin Capsules: Potential Materials for Packaging Films. Ind. Crops Prod..

[148] Medina-Jaramillo C., Ochoa-Yepes O., Bernal C., Famá L. (2017). Active and Smart Biodegradable Packaging Based on Starch and Natural Extracts. Carbohydr. Polym..

[149] Pagnan C.S., Mottin A.C., Oréfice R.L., Ayres E., Câmara J.J.D. (2018). Annatto-Colored Poly(3-Hydroxybutyrate): A Comprehensive Study on Photodegradation. J. Polym. Environ..

